# tasiR-ARFs Production and Target Regulation during In Vitro Maize Plant Regeneration

**DOI:** 10.3390/plants9070849

**Published:** 2020-07-06

**Authors:** Brenda Anabel López-Ruiz, Vasti Thamara Juárez-González, Andrea Gómez-Felipe, Stefan De Folter, Tzvetanka D. Dinkova

**Affiliations:** 1 Departamento de Bioquímica, Facultad de Química, Universidad Nacional Autónoma de Mexico, 04510 Ciudad de Mexico, Mexico; lopezruizba@comunidad.unam.mx (B.A.L.-R.); vasti.juarez.gonzalez@gmail.com (V.T.J.-G.); 2 Centro de Investigación y de Estudios Avanzados del Instituto Politécnico Nacional (CINVESTAV-IPN), Unidad de Genómica Avanzada (UGA-LANGEBIO), 36821 Irapuato Gto., Mexico; andrea.gomez@cinvestav.mx (A.G.-F.); stefan.defolter@cinvestav.mx (S.D.F.)

**Keywords:** auxin response factor, callus, de novo shoot regeneration, maize, tasiR-ARF

## Abstract

During in vitro maize plant regeneration somatic cells change their normal fate and undergo restructuring to generate pluripotent cells able to originate new plants. Auxins are essential to achieve such plasticity. Their physiological effects are mediated by auxin response factors (ARFs) that bind auxin responsive elements within gene promoters. Small trans-acting (ta)-siRNAs, originated from miR390-guided *TAS3* primary transcript cleavage, target ARF3/4 class (tasiR-ARFs). Here we found that *TAS3b* precursor as well as derived tasiR-ARFbD5 and tasiR-ARFbD6 display significantly lower levels in non-embryogenic callus (NEC), while *TAS3g*, miR390 and tasiR-ARFg are more abundant in the same tissue. However, Argonaute (AGO7) and leafbladeless 1 (LBLl) required for tasiR-ARF biogenesis showed significantly higher transcript levels in EC suggesting limited tasiR-ARF biogenesis in NEC. The five maize ARFs targeted by tasiR-ARFs were also significantly enriched in EC and accompanied by higher auxin accumulation with punctuate patterns in this tissue. At hormone half-reduction and photoperiod implementation, plant regeneration initiated from EC with transient *TAS3g*, miR390 and tasiR-ARFg increase. Upon complete hormone depletion, *TAS3b* became abundant and derived tasiR-ARFs gradually increased at further regeneration stages. *ZmARF* transcripts targeted by tasiR-ARFs, as well as *AGO7* and *LBL1* showed significantly lower levels during regeneration than in EC. These results indicate a dynamic tasiR-ARF mediated regulation throughout maize in vitro plant regeneration.

## 1. Introduction

Cereals like maize are the most important food crops in the world. In vitro tissue culture has proven to be an important tool for genetic improvement, massive propagation and transformation of these species. During this process, induction and regeneration of embryogenic callus (EC) depends on the genotype, explant and establishment of appropriate stimuli [1,2].

While many dicotyledonous plants can be regenerated from leaf material, monocotyledonous, especially the family Poaceae to which maize belongs, are difficult to propagate and tend to be recalcitrant for plant regeneration. Preferred monocot explants used for EC induction are immature embryos [3]. Several maize inbred lines producing EC are able to regenerate plants via shoot organogenesis or somatic embryogenesis. Such is the case of the Mexican improved variety VS-535 developed by Instituto Nacional de Investigaciones Forestales, Agricolas y Pecuarias (INIFAP) from the Tuxpeño landrace genotype [4,5]. The precise mechanisms underlying efficient dedifferentiation, embryogenic potential acquisition and proliferation establishment for callus tissues are unknown, but auxin and cytokinin interplay exerts a major role within the process. These two phytohormones control cell proliferation and differentiation by establishing antagonistic signals in meristematic cells. While auxin is required to induce and maintain dedifferentiation from the explant, including small amount of cytokinin would promote the proliferation status of the induced callus and keep its pluripotency [6].

According to recent reports, transcription factors targeted by small RNAs (sRNAs) are implicated in the dedifferentiation and redifferentiation process [7,8]. sRNAs originate from single-stranded self-complementary hairpin structures (microRNAs (miRNAs)) or from double-stranded RNA (dsRNA) precursors (small interfering RNAs (siRNAs)) [9]. In some cases, transcripts targeted by a miRNA can trigger the production of secondary siRNAs in phase (phasiRNAs) such as the trans-acting tasiRNAs targeting RNAs distinct from their original precursors [10,11].

tasiRNAs biogenesis is characterized by the initial precursor (*TAS* transcript) recognition and cleavage, directed by specific miRNA charged on an Argonaute (AGO7) protein. The event triggers further double stranded RNA (dsRNA) production by RNA-dependent RNA polymerase 6 (RDR6), suppressor of gene silencing 3 (SGS3) corresponding to leafbladeless 1 (LBL1) in maize [12] and dsRNA binding 4 (DRB4) [13]. The dsRNA is sequentially processed by Dicer-like 4 (DCL4) into 21-nucleotide tasiRNA duplexes in phase with the miRNA guided cleavage site [10,14]. One strand of the tasiRNA duplex is selectively loaded on particular AGO according to the sRNA 5′ nucleotide [15,16].

Four TAS gene families, *TAS1*, *TAS2*, *TAS3* and *TAS4*, are present in *Arabidopsis thaliana*. *TAS1* and *TAS2* precursors are subject to miR173 cleavage, while *TAS3* and *TAS4* are recognized by miR390 and miR828, respectively [17]. *TAS3* transcript bears two target sites for miR390, loaded specifically on AGO7, and generates tasiRNAs via the “two-hit” mechanism [14]. However, it has been shown that a single miR390 targeting event is sufficient for *TAS3*-based tasiRNA biogenesis [18]. Only *TAS3* derived tasiRNAs are widespread suggesting that these regulatory RNAs belong to an evolutionary conserved pathway in terrestrial plants [19]. tasiRNAs target members of the auxin response factor (ARF) transcription factor family ARF3/4. Thereafter, they were termed tasiR-ARFs [20].

tasiR-ARFs participate in diverse developmental processes, organ patterning, germination and in response to abiotic stress [21,22,23,24,25,26]. However, their role in *de novo* shoot apical meristem (SAM) formation and in vitro plant regeneration is unclear. Components of the tasiR-ARF pathway were explored in *Dimocarpus longan* somatic embryo development indicating that miR390 levels are lower in embryogenic callus and higher in torpedo-shaped embryos. Additionally, *TAS3* and *ARF4* transcripts exhibited their lowest abundance in the EC, peaked in globular embryos and dropped at the torpedo stage, inversely proportional to miR390 at this stage [27]. In *Larix leptolepis*, *TAS3* depicted inverse correlation with high miR390 and tasiR-ARFs levels in cotiledonary somatic embryos [28,29]. On the other hand, miR390 was abundant at early globular-shaped embryo formation in *Citrus sinensis* [30] and *Gossypium hirsutum* EC [31]. These data suggest that expression patterns of tasiR-ARFs pathway components during in vitro tissue culture differ, depending on the plant species, albeit their presence and impact on ARF3/4 regulation could be relevant for the regeneration process.

Here we explored the transcript level dynamics of maize *TAS3* precursors and other components of the tasiR-ARF pathway during dedifferentiation and in vitro plant regeneration of Tuxpeño VS-535 cultivar. The most abundant precursors, *TAS3b* and *TAS3g*, derived tasiR-ARFs and five ARF3-like *ZmARF* targets were analyzed in EC and in non-embryogenic callus (NEC), and at different regeneration stages. Established NEC presented significantly lower levels for all tasiR-ARF pathway components with respect to the EC, except for miR390, *TAS3g* and tasiR-ARFg. Plant regeneration was characterized by a drop in transcript abundance for *AGO7*, *LBL1* and ARF3-like *ZmARF* transcripts, whereas *TAS3b*, *TAS3g* and derived tasiR-ARFs showed non-overlapping accumulation patterns at the different stages. Auxin distribution was different between EC and NEC, as well as at regenerative spots, supporting the differential role of tasiR-ARF pathway in these tissues. According to our results we propose that active tasiR-ARF mediated regulation is present in the EC, but not maize NEC tissues, to maintain the dedifferentiated status and embryogenic potential in response to high exogenous auxins. Upon plant regeneration promotion, the pathway might be required to achieve ARF transcript downregulation and/or re-localization at early differentiation stages.

## 2. Results

### 2.1. Maize TAS3 and tasiR-ARF Components Gene Expression

All analyses were performed with maize calli induced from Tuxpeño VS-535 immature embryos. After subcultures for 8–12 months on N6 proliferation medium (see methods) calli were separated in embryogenic (EC) and non-embryogenic (NEC) types according to their morphology (Supplementary Materials Figure S1; [32]). For plant regeneration, phytohormones were gradually depleted under a photoperiod. The first stage was represented by regenerative spots obtained on half-concentration hormones in N6, the second stage were leaf-resembling structures produced at two additional weeks on N6 without hormones and the third stage was characterized by differentiated leaf, still adhered to the callus, after two weeks on Murashige and Skoog (MS) medium. Plantlet was considered the final stage when rooting and full leaf development was observed on MS medium. No regeneration was observed from NEC (Supplementary Materials Figure S1). According to this procedure, shoot organogenesis was the predominant way of plant regeneration, but also somatic embryogenesis (SE) has been reported for Tuxpeño VS-535 [5].

In maize, the *TAS3* loci family comprises seven *TAS3* genes that generate mature tasiR-ARFs. According to transcriptomic data available in our lab, while in the immature maize embryo most *TAS3* genes are expressed, their transcript levels are strongly reduced upon callus induction [33]. Therefore, as an initial approach we analyzed the transcripts produced from *TAS3 a–e*, *g* and *i* genes by endpoint RT-PCR (Supplementary Materials Figure S2). While *TAS3g* was present in EC, NEC and all regeneration stages, *TAS3i* was not detected in any tissue. The other transcripts showed differential patterns depending on the sample. *TAS3d* and *TAS3e* transcripts were observed in NEC, but not in EC. During regeneration, expression of *TAS3b* and *TAS3c* was observed at the first stage. *TAS3b* showed reduction at the second and increment third stages, whereas *TAS3c* remained present until plantlet. *TAS3a* appeared at the third stage and *TAS3e* decreased during regeneration. All *TAS3* transcripts were present in the regenerated plantlet, except *TAS3i*. This precursor was previously found in the maize immature embryo, but sharply decreased to undetectable levels upon callus induction [33].

The maize genome encodes 38 ARFs, from which 37 were previously detected at transcript level during callus induction [33]. Some of them increased, while others decreased their expression within the process. As described for Arabidopsis, maize ARFs were grouped within six classes [34] and Class I includes ARF3/4-like members targeted by tasiR-ARFs. From these *ARF* transcripts, only *ZmARF11*, *ZmARF12* and *ZmARF24* were amplified from all samples. *ZmARF23* was detected only in NEC, while *ZmARF26* was present in EC. Although final point RT-PCR is not quantitative, a decrease in the level of some *ZmARFs* was observed during regeneration. Maize miR390 has two precursors: *MIR390A* and *MIR390B*, but only the transcript corresponding to *MIR390A* was observed. These results indicated that components of the tasiR-ARF pathway are present during maize in vitro culture and are probably differentially accumulated throughout the regeneration process. We selected *TAS3b*, *TAS3g* transcripts and derived tasiR-ARFs: tasiR-ARFbD5, tasiR-ARFbD6 and tasiR-ARFg for further qPCR analysis. Together these tasiR-ARFs are able to target the five ARF3-like *ZmARF* mRNAs (Supplementary Materials Figure S3). Transcripts corresponding to the unique *LBL1* and *AGO7* maize genes, as well as mature miR390 and the five *ARF* targets were also tested to either compare EC and NEC as dedifferentiated tissues with contrasting regenerative potential, or to appreciate dynamics of the tasiR-ARF pathway during the defined plant regeneration stages.

### 2.2. Differential Abundance of tasiR-ARF Pathway Components in EC and NEC Calli

**Figure 1:**
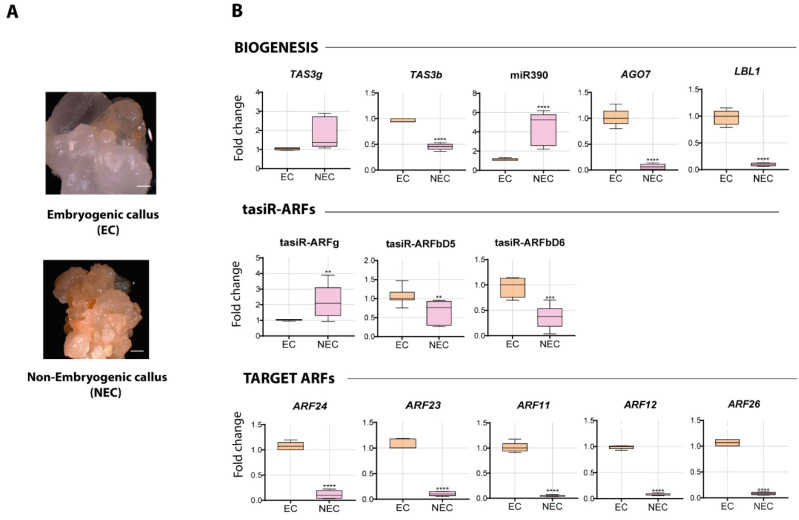
Differential accumulation of tasiR-auxin response factors (ARF) pathway components in embryonic callus (EC) and non-embryonic callus (NEC). (**A**) callus types observed during subcultures of Tuxpeño VS-535: EC and NEC. (**B**) transcript levels were analyzed by RT-qPCR in established 12 months-old EC and NEC. Upper charts depict the elements involved in tasiR-ARFs biogenesis. Middle panel shows mature tasiR-ARFs derived from *TAS3b* and *TAS3g* precursors. Bottom charts indicate the abundance of *ZmARFs* from the ARF3 family in maize targeted by tasiRNAs. Fold change (FC) represents abundance relative to the EC and normalized by U6 snRNA internal control for tasiR-ARFs or 18S rRNA for long transcripts. The results were obtained from two independent biological replicates (*n* = 2) with three technical replicates for each one (*n* = 6). Student’s t-test was used to evaluate if statistically significant differences existed between EC and NEC transcripts levels. Significant values are indicated as follows: (*) *p* < 0.05, (**) *p* < 0.01, (***) *p* < 0.001 and (****) *p* < 0.0001.

Proliferating Tuxpeño VS-535 maize EC and NEC (Figure 1A) were compared for tasiR-ARF pathway transcript accumulation in 12 months-old subcultures. While the *TAS3g* precursor, miR390 and tasiR-ARFg were significantly more abundant in NEC, *TAS3b* precursor and derived tasiR-ARFs, as well as transcripts corresponding to *AGO7* and *LBL1*, functionally demonstrated to be required for tasiR-ARF production in maize, showed higher levels in EC (Figure 1B). While not all enzymes participating in tasiR-ARF biogenesis and function were analyzed, these results suggest that key components might be limiting for *TAS3* precursor processing in NEC. Strikingly, all *ZmARF* targets showed significantly higher levels in EC, where the pathway seems to be active. One possible explanation might reside on different patterns of auxin distribution in EC and NEC resulting in a positive response only for EC. It has been proposed that tasiR-ARF regulation enhances the robustness of auxin responses by limiting ARF transcription factors reported to act as repressors (ARF3/4), in tissues where their expression has been stimulated [35]. Hence, a contrasting tasiR-ARF activity between calli with different embryogenic potential might support appropriate auxin responses in the EC and maintenance of its regenerative ability upon auxin depletion.

### 2.3. Readjustment of tasiR-ARF Pathway Component Accumulation Patterns During Plant Regeneration

**Figure 2:**
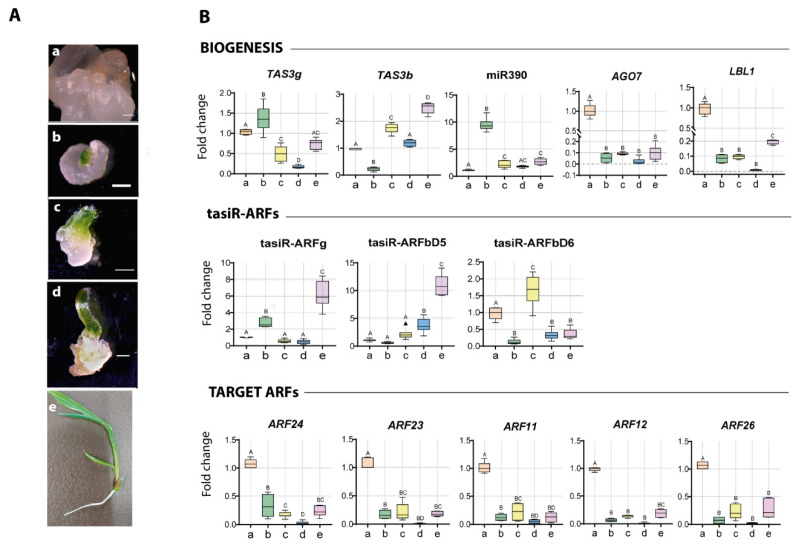
In vitro maize regeneration is accompanied by increase in tasiR-ARF production and *ZmARF3* transcripts down-regulation. (**A**) stages of in vitro regeneration of Tuxpeño VS-535: (a) EC, (b) 1st stage of development, (c) 2nd stage of development, (d) 3rd stage of development and (e) plantlet. (**B**) tasiR-ARF pathway components abundance was analyzed by RT-qPCR at each regeneration stage. Upper charts depict the elements involved in tasiR-ARFs biogenesis. Middle panel shows mature tasiR-ARFs derived from *TAS3b* and *TAS3g* precursors. Lower charts indicate the abundance of *ZmARFs* from the ARF3 family in maize targeted by tasiRNAs. Fold change represents abundance relative to the EC and normalized by U6 snRNA internal control for tasiR-ARFs or 18S rRNA for long transcripts. The results were obtained from two independent biological replicates (*n* = 2) with three technical replicates for each one (*n* = 6). For multiple comparisons, data were subjected to one-way analysis of variance (one-way ANOVA) and differences between the means were compared by the Tukey post-hoc test. Bars that do not share at least a common letter differ significantly (*p* < 0.05).

In plant regenerative spots (Figure 2A, first stage), *TAS3g* precursor, tasiR-ARFg and miR390 increased with respect to the non-differentiated tissues, while *AGO7*, *LBL1* and all *ZmARF* targets sharply decreased (Figure 2B; lanes b). The simultaneous miR390 and tasiR-ARFg increments suggest *TAS3g* precursor processing occurs around this stage. During the second stage of development, characterized by a subtle rolled leaf shape tip (Figure 2A), a boost of *TAS3b* precursor and one of its derived mature tasiRNAs (tasiR-ARFbD6) were observed (Figure 2B, lanes c). However, mature miR390 levels significantly dropped at this stage. For the third stage, most components showed very low levels, except for *TAS3b* and the corresponding tasiR-ARFs (Figure 2B, lanes d).

In the fully regenerated plantlet, transcripts for many components of the tasiR-ARF pathway increased. Particularly, a significant production of *TAS3b* and *TAS3g* precursors, tasiR-ARFg, tasiR-ARFbD5 and *LBL1* was evident. *ZmARF12*, *ZmARF23* and *ZmARF24* also appeared significantly more abundant. Interestingly, the increment of tasiR-ARFbD5 was not mirrored by tasiR-ARFbD6, in spite of being derived from the same precursor. This might result from either processing or sRNA stability, or could be further affected by additional components, such as DCL4 and AGO1.

**Figure 3:**
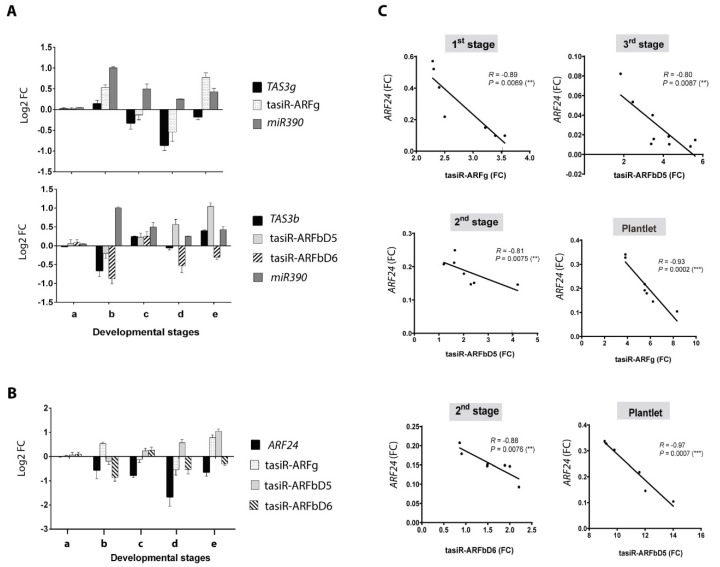
Correlation between accumulation patterns of precursors, tasiR-ARFs and the *ZmARF24* target. (**A**) inverse correlation (IC) between *TAS3* transcripts, tasiR-ARFs and miR390 levels. *TAS3g* shows IC with miR390 abundance, and tasiR-ARFg increment in the plantlet. Similarly, *TAS3b* inversely mirror a boost of tasiR-ARFbD5 levels at the 3rd stage and plantlet. (**B**) IC between tasiR-ARFs and *ZmARF24*. *ZmARF24* levels inversely correlate with distinct tasiR-ARFs depending on the developmental stage: (a) EC, (b) 1st stage, (c) 2nd stage, (d) 3rd stage and (e) plantlet. (**C**) the negative correlation between *ZmARF24* and particular tasiR-ARFs was validated by Pearson’s correlation coefficients at different regeneration stages. Pearson analysis was performed using the FC values. FC was calculated using the 2^−∆∆ct^ method, considering EC tissue as reference and rRNA 18s or U6 snRNA as internal housekeeping controls. The R and *p* value are indicated on each graph.

Furthermore, we detected an inverse correlation between *TAS3* transcripts, mature tasiRNAs and miR390 at some stages, which suggests active *TAS3* processing and tasiRNA production. Particularly, an increase of tasiR-ARFg and miR390 abundance correlated with a decrease of *TAS3g* levels in plantlet. Similarly, *TAS3b* negatively correlated with tasiR-ARFbD5 levels at the third stage and plantlet (Figure 3A).

The levels of the five *ZmARFs* inversely correlated with different tasiRNAs depending on the regeneration stage (Figure 3B and Supplementary Materials Figure S4). For example, at the first stage of regeneration inverse correlation was observed for tasiR-ARFg, while at the second and third stages tasiR-ARFbD5 levels negatively correlated with *ZmARF24* transcript. The inverse relationships between different tasiRNAs and *ZmARF24* were validated by Pearson correlations, displaying satisfactory parameters for negative correlation (Figure 3C).

Overall, these observations indicated that tasiR-ARF regulation operates during maize in vitro plant regeneration and is particularly relevant to restrict the activity of ARF3 family since early differentiation stages. Auxin signaling is crucial for both, dedifferentiation and differentiation processes during in vitro culture, and the hormone localization within the callus masses has been found as crucial for regenerative spot delimitation [36]. To explore its role in maize in vitro plant regeneration we analyzed the spatio-temporal pattern of the phytohormone in EC, NEC and at the first stage of regeneration.

### 2.4. Immunolocalization of Auxin

**Figure 4:**
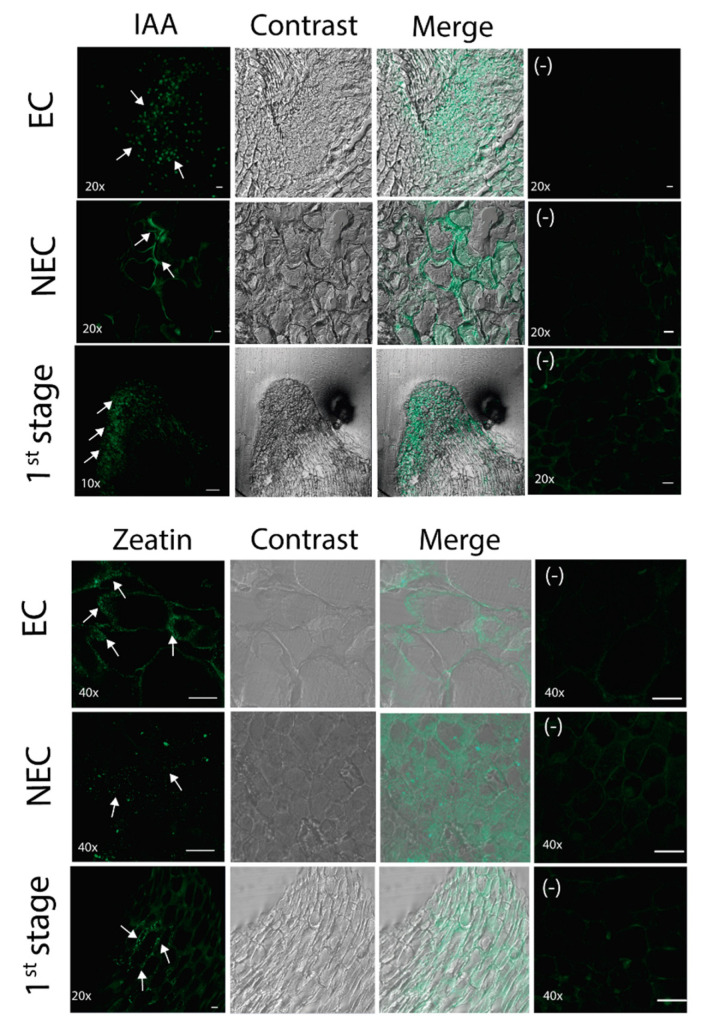
Immunolocalization of auxin and cytokinin in different tissues. Sections from NEC, EC and the 1st regeneration stage were analyzed using anti-indole-3 acetic acid (IAA) and anti-trans zeatin antibodies. Fluorescence, bright field and merged images are shown for each tissue. Microscope magnification and negative control (samples incubated without primary antibody) are represented in the images. The arrows indicate fluorescence signal. In the NEC, IAA exhibited more intense signal at cell periphery, while in the EC, the auxin was detected as interspersed spots. During the 1st stage of regeneration, the auxin signal was detected at the tip of the regenerative spot. The cytokinin immunolocalization was observed only for EC tissue, as patches surrounding cells, and at the 1st stage of development over the middle portion of a regeneration spot. Bar = 20 µm, except in the auxin immunolocalization from 1st stage, where bar = 100 µm.

Immunolocalization was performed with an antibody developed against indole-3 acetic acid (IAA), which is a naturally occurring auxin. IAA localization depends mostly on its biosynthesis and transport, and together with cytokinins, it plays important roles in cell division control [37]. At the stage of de-differentiated tissues IAA distribution was different for EC and NEC (Figure 4, upper panels). In NEC, the IAA signal was low and occasionally more intense at cell periphery, surrounding large cells commonly observed for this tissue. Conversely, IAA was detected as abundant punctuated spots in or at the edge of small cells characteristic to meristematic zones of the EC tissue. Such different patterns of IAA suggests auxin transport, and probably signaling, are different between EC and NEC. Supporting this, PIN FORMED (PIN) expression was only detected in the EC (Supplementary Materials Figure S5). During the first stage of regeneration, a strong auxin signal was detected at the tip of the regenerative spot with its maximum at the edge of the tissue. This pattern suggests that upon external 2,4-D depletion, there is an auxin gradient establishment within the EC, which might be required for leaf primordium morphogenesis.

### 2.5. Immunolocalization of Cytokinin

It is well known that auxin and cytokinin-mediated regulatory pathways establish cross-talks for cell type specialization during maize organ formation [38]. While auxin participates in lateral primordium formation, cytokinin promotes meristem cell proliferation. Besides, during the de novo shoot regeneration the local auxin distribution determines the cytokinin response, which is partially mediated by ARF3 [39]. The localization of this phytohormone was tested in de-differentiated tissues and at the first regeneration stage using an antibody against trans-zeatin (a natural cytokinin). While a weak signal was observed in NEC, a stronger signal was observed in EC as patches surrounding cells within the tissue (Figure 4, bottom panels). There was a slightly stronger signal at one side of each cell. The first stage of regeneration featured lower cytokinin signal at particular region within the middle portion of the regenerative spot. The localization of signal near the edge of cells may be indicative of apoplastic cytokinin, suggesting non-overlapping localization with auxin in either de-differentiated tissues or at early plant regeneration stages.

## 3. Discussion

The phytohormone auxin is a major regulator in plant development; its function is exerted through diverse signaling pathways and particular localization within plant tissues is governed by biosynthesis, metabolism and transport [40]. Signaling is mediated by ARFs that bind to auxin responsive elements (AuxRE) within gene promoters to orchestrate particular expression programming according to finely established auxin concentration gradients [41,42,43]. *Arabidopsis* ARF3, also known as ETTIN or ETT, is atypical because of its lack of interaction with Aux/IAA repressors due to the absence of a Phox and Bem1 (PB1) domain at the carboxi terminus [44,45]. ARF3 functions as a non-canonical auxin sensor, where its C-terminal ETT-Specific (ES) domain is able to bind auxin directly, resulting in interference with protein–protein interactions with different transcription factors [46]. Therefore, ARF3/4 regulation by tasiRNA-ARFs has emerged as an important evolutionary conserved mechanism to fine-tune auxin concentration sensitivity during development [19,35].

The biogenesis of tasiR-ARFs is important for maize organ specification [12] and the mir390-ARF3/4 module controls lateral root development in Arabidopsis [23], which share some genetic similarity with callus formation via pericycle-like cells [47,48]. However, their specific roles during the tissue dedifferentiation process, callus formation, de novo organogenesis and somatic embryogenesis have not been explored. Considering their relevance for plant organ development, tasiR-ARFs could relate to callus embryogenic capacity and successful plant regeneration. Moreover, in spite of lack of clear connection between tasiR-ARFs and the tissue dedifferentiation process, changes in the abundance of several pathway components have been related to the embryogenic potential of callus in maize [33].

Here we report different accumulation patterns for most tasiR-ARF pathway elements between calli with contrasting embryogenic potential and during in vitro maize regeneration stages. Non-embryogenic callus was characterized by accumulation of *TAS3g*, tasiR-ARFg and miR390. This is in line with previously described high miR390 levels in NEC from other plant species, such as citrus [49] and Arabidopsis [50]. However, miR390 was also reported as upregulated in EC and during the formation of somatic embryos in cotton [31] and rice [51]. Hence, depending on the plant species and the in vitro culture process, miR390 and tasiR-ARFs might contribute differently to the embryogenic potential.

The maize embryogenic callus notably accumulated *TAS3b*, tasiR-ARFbD5/6, *LBL1*, *AGO7* and the five transcripts from ARF3 family, *ZmARF11*, *12*, *23*, *24* and *26*, targeted by tasiRNAs. This callus can be considered as a mass of pluripotent cells with meristematic-like features. *TAS3b* transcript expression has been reported for the tip of maize SAM (L1 and L2 layers), where tasiR-ARFs could be produced but not accumulated [12]. Moreover, LBL1 and AGO7 are required for shoot development since *lbl1-rgd/rgd2-R* double mutant kernels develop embryos which form normal root pole but no SAM, coleoptile or leaf primordia [24]. All these evidences strongly suggest that LBL1, AGO7 and *TAS3b* participate in SAM formation and feasibly confer to this niche their features as meristematic cells prone to differentiation of aerial organs. Additionally, LBL1 might be related to the regulation of transposons, particularly the *gyma* class of long tandem repeat (LTR)-retrotransposons [20]. Therefore, the highest accumulation of *LBL1* transcript in the EC could help to maintain genome integrity required for a successful regenerative process.

According to auxin distribution and *ZmARF3*-like expression, maize EC elicits greater response to auxin than NEC. In Arabidopsis, *ARF3* expression is induced by the auxin rich medium that leads to callus formation and is a key factor during *de novo* shoot regeneration [39]. In our model, the five *ZmARF3*-like genes were highly expressed in the EC, perhaps due to the elevated concentrations of exogenous hormones in the culture medium and enhanced distribution of the phytohormone in this tissue. Besides, such accumulation of ARF3-like family members in EC might be relevant for appropriate transmission of the IAA signal, which is also higher for this tissue. Therefore, interaction between these ARFs and other transcription factors operating in response to auxins, together with the ability to establish auxin polar transport (PIN expression was apparently exclusive to EC; Supplementary Materials Figure S5) could be leading to special features of a pluripotent tissue.

**Figure 5:**
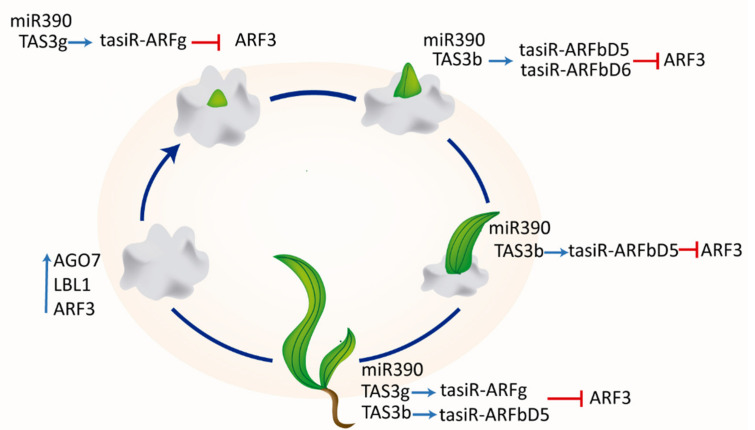
tasiR-ARFs pathway dynamics during maize in vitro plant regeneration. Maize VS-535 regeneration takes place via leaf formation on the callus surface and the established stages display differential accumulation of selected tasiR-ARF to downregulate the transcripts of ARF3-like ZmARF family. At some stages, the elevated tasiRNA levels correspond to decrement of *TAS3* precursor and targeted *ZmARFs*, as well as increase of miR390. At the 1st stage, characterized by regenerative spot formation, there is IC between tasiR-ARFg and ARF3-like *ZmARFs*. The apical growth of the green spot resembling leaf tip structure at the 2nd stage corresponds to IC between *TAS3b*-derived tasiRNAs and *ZmARF* targets. At the 3rd stage, represented by typical leaf development still adhered to the callus, ARF3-like *ZmARF* transcripts negatively correlate with tasiR-ARFbD5. In regenerated plantlet, there is an IC between tasiR-ARFg, tasiR-ARFbD5 and ARF3-like *ZmARF* transcripts.

Early regeneration in our system represents leaf primordium formation and specification of its polarity is supported by miR390 and tasiRNAs generation. At the same stage, we observed the highest transcript levels for KNOTTED1 (KN1; Supplementary Figure S5), orthologue of SHOOT MERISTEMLESS (STM) in Arabidopsis, that confers meristematic identity and is the first marker available in maize to monitor formation of the SAM during embryogenesis [52]. WUSCHEL related homeobox 3A (WOX3A) also increased at this stage, suggesting the beginning of leaf primordium formation (Supplementary Materials Figure S5). This gene expression marks a ring-shaped domain of cells recruited for the P0/P1 leaf primordium and is detected at the margins of developing true foliage leaves [53]. Notably, its transcript was detected in EC, but not in NEC, supporting different molecular features of the tissues. These results suggest that SAM and leaf primordium formation programs are available for the same tissue where tasiR-ARFs could help to establish leaf polarity since early regeneration (Figure 5). Increase of tasiR-ARFg, but not tasiR-ARFbD5/6 during the first stage might reflect precursor availability, since *TAS3g* abundance is greater than any other *TAS3* in calli (Supplementary Materials Figure S1). While the regulation supporting differential tasiRNA species accumulation is unknown, it could also result from cell type-dependent gradients. The movement of tasiR-ARFs from its well-defined source of biogenesis has been described as the origin of known concentration gradients dissipating abaxially [54] to allow a sharply defined domain of ARF3 expression on the abaxial side of leaf primordia [55,56].

The transcripts decrease of the five *ZmARF3*-like genes observed upon regeneration induction is also part of the differentiation process, allowing their re-localization to particular cell types in the regenerating tissues. Consistent with ARF3 role in regeneration responses, Arabidopsis *arf3* mutants showed reduced SE induction [57] and shoot regeneration, while none of the other ARF repressors affected the frequency of *de novo* shoot formation [39]. In the same model, responses to auxin levels control the spatiotemporal distribution of cytokinin biosynthesis through decreasing expression of ATP/ADP isopentenyl-transferase5 (*IPT5*) by repressor ARF3 bound to its promoter. Such auxin-cytokinin crosstalk is critical for the spatially restricted WUSCHEL (WUS) induction required for shoot meristem establishment and regeneration [39].

Overall, this study shows that in vitro maize regeneration is characterized by differential accumulation of selected tasiR-ARF species to provide appropriate downregulation of their targets belonging to the ZmARF3 family (Figure 5). In such a way, the tissues may elicit developmental responses when endogenous auxin redistributes. The current panorama on specific EC features depicts greater accumulation of tasiR-ARF biogenesis components and *ZmARF3* transcripts, expression of PIN auxin polar transporters and enrichment in auxin response-related miRNAs and targets [32]. This information provides insights into the genetic program underlying *de novo* plant formation, where leaf initiation and morphogenesis appear as primary and essential processes.

## 4. Materials and Methods

### 4.1. Plant Materials, Culture Conditions and Sample Collection

Immature embryos from Mexican cultivar VS-535 were collected at 15–18 days after pollination of greenhouse-cultured plants. The callus induction, subculture conditions and plant regeneration were described in detail in a previous study [32]. The callus tissues were subcultured for 8–12 months before sampling and plant regeneration induction.

### 4.2. RNA Isolation

RNA was isolated from EC, NEC and four previously established plant regeneration developmental stages [32] in two biological samples. RNA sized fractionation to large (>200 nt) and small (<200 nt) RNAs was performed according to [58] The RNA quality and concentration were determined using the NanoDrop Spectrophotometer (Thermo Fisher Scientific, Waltham, MA, USA).

### 4.3. qRT-PCR

Reverse transcription (RT) for large RNAs, was carried out using an oligo (dT) primer and the ImProm-II™ reverse transcription system (Promega, Madison, WI, USA). Specific primers for *TAS3*, *AGO7*, *LBL1* and *ARF3* transcripts were designed using Primer-BLAST optimized for real-time PCR (qPCR). The amplification products of *TAS3* included the miR390-binding site and the tasiRNAs originating sequence. The *ARF3* maize transcripts targeted by tasiR-ARF were selected according to [20]. These included *ZmARF11* (*arf3c*), *ZmARF12* (*arf3d*), *ZmARF23* (*arf3b*), *ZmARF24* (*arf3a*) and *ZmARF26* (*arf3e*); the nomenclature in parenthesis is the one used in [20]. The amplification products included the predicted tasiRNA-directed cleavage site. For small RNAs, the stem-loop RT and forward primers were designed according to [59]. All oligonucleotide sequences used in this study are available in Supplementary material (Supplementary Materials Table S1).

Pulsed stem-loop RT was performed for three tasiR-ARFs, miR390 and the U6 snRNA as control in duplicate reactions [58]. For qPCR, the Maxima SYBR Green/ROX qPCR Master Mix and the 7500 Real-time PCR System (Applied Biosystems, USA) were used. Abundances were expressed as “fold change” (FC) using the 2^−∆∆^Ct method, considering the EC tissue as reference and rRNA 18S or U6 snRNA as internal housekeeping controls for large and small RNAs, respectively. Results were averaged for biological and technical replicates, 6–12 independent measurements. Statistical analyses were performed using t-student and one-way ANOVA method. The association between tasiR-ARFs and *ZmARF24* transcript levels was tested by Pearson´s correlation coefficients using the FC values.

### 4.4. Auxin and Cytokinin Immunolocalization

Tissues corresponding to EC, NEC and the first stage of regeneration were fixed using 3% paraformaldehyde and 0.5% glutaraldehyde in 1% microtubule-stabilizing (MTBS) buffer (2x MTBS: 15 g 1,4-Piperazinediethanesulfonic acid (PIPES), 1.9 g glycol ether diamine tetraacetic acid (EGTA), 1.22 g MgSO_4_.7H_2_O and 2.5 g KOH, pH 7.0) with 0.1% Triton X-100. The ratio was 10 volumes of fixative to 1 volume of tissue. Tubes were placed in a vacuum desiccator and vacuum was applied for 30 min. Afterwards, samples were incubated at 4 °C for 2.5 h. The tissues were rinsed with distilled water for 10 min. The samples were dehydrated by passing through a series of ethanol solutions (20%, 30%, 50%, 70%, 80%, 90% and 100% ethanol); 1 h each, at 4 °C. Samples were embedded in the resin Technovit according to manufacturer instructions (Heraeus Kulzer, Hanau, Germany). Blocks were sectioned using a microtome; sections were 12–18 µm thick. The quality of the sections was checked by placing them on a glass slide with water, after that, 5–10 sections were transferred to one well (24-well plate) with 1 mL distilled water (free floating of tissue sections). Later, the samples were treated three times for 10 min each with blocking buffer (2% albumin fraction (BSA) in 1% MTBS buffer). The tissues were incubated with 1% of the primary antibody (1:100 dilution; anti-indole-3-acetic-acid or anti-trans-zeatin riboside; Olchemim, Olomouc, Czech Republic) in blocking buffer and 0.025% Tween 20 at 4 °C overnight. Then, the primary antibody was washed with 1% MTBS buffer two times for 5 min. The samples were incubated 1 h with secondary antibody DyLightR 488 (1:200 dilution; Abcam, Cambridge, United Kingdom) in blocking buffer. The tissues were washed with 1% MTBS buffer three times for 5 min. The floating sections were transferred to glass slides and observed immediately under confocal microscopy LSM 800 (Zeiss, Oberkochen, Germany). Signal was visualized by excitation with a 488 nm diode laser (0.2–0.5% laser power), emission collected between 489 and 545 nm, at a resolution of 1024 × 1024 pixels.

## Supplementary Materials

The following are available online at https://www.mdpi.com/2223-7747/9/7/849/s1, Table S1. Oligonucleotides used in this study. Figure S1. In vitro maize regeneration process. Figure S2. *TAS3*, *ARF3*, and *MIR390* transcript analysis during in vitro maize plant regeneration. Figure S3. tasiR-ARF and ARF3-like *ZmARF* target sequences. Figure S4. Stage-dependent inverse correlation between tasiR-ARFs and *ZmARF* targets. Figure S5. RT-PCR analysis of genes involved in maize SAM and leaf establishments.
