# Supplementary material for: tasiR-ARFs Production and Target Regulation during In Vitro Maize Plant Regeneration
Source: Plants (Basel). 2020 Jul 6;9(7):849. doi: 10.3390/plants9070849 (PMC7411845; doi:10.3390/plants9070849)
Supplement: Supplementary file 1 [file plants-09-00849-s001.pdf]

Supplementary Information for:

tasiR-ARFs production and target regulation during in vitro maize plant regeneration

Brenda A. López-Ruiz[1], Vasti T. Juárez González[1], Andrea Gómez-Felipe[2], Stefan de Folter[2], Tzvetanka D. Dinkova[1]*

Table S1. Oligonucleotides used in this study. miRNA sequences were retrieved from miRBase (http://www.mirbase.org) and tasiRNAs sequence from Dotto et al., 2014. Target cDNA sequences from Maize Genetics and Genomic Database (https://www.maizegdb.org/).

ID		SECUENCE (5´→3´)
**TAS3a**	GRMZM2G178686	Forward GTAAGGCCTCTTCTTGACCTTGTA<br>Reverse CACAGGGTGAAAACATTAACTGAAC
**TAS3b**	GRMZM2G020468	Forward CTTGACCTTGTAAGACCCAACTCTA<br>Reverse TGTTTGTCTCATGCCTCACTCTAT
**TAS3c**	GRMZM2G084821	Forward GGTTCACGTGGTTCATGTCTAGTAT<br>Reverse AAACATATAGTTGAACCCACAGC
**TAS3d**	GRMZM2G124744	Forward GGTTTCTCGTGCCAGAATTAAC<br>Reverse AATAATTTCAACGCCACCAAAC
**TAS3e**	GRMZM5G806469	Forward TGATTGACGCTATCCTCGTCG

		Reverse AGGACTGTAGGAGTCGCAGG
**TAS3g**	GRMZM2G082055	Forward ACCTATTCACCACCGCTGTC<br>Reverse TGCGAGAGTGTTCCAAGCTC
**TAS3i**	GRMZM2G512113	Forward TTCGCAGTCTTTGTTTCATCA<br>Reverse AGCGGTACAAGCTCAAGAGG
**ARF24**	GRMZM2G030710	Forward CTTCCCCATGTTAATCCAGACTAC<br>Reverse CAGCAGCATTGTCATGAGTTCTAT
**ARF23**	GRMZM2G441325	Forward AAGCTACTTATGCTGTCTGCTGTG<br>Reverse TCTGGTTCCAACTGAAAAGTGATA
**ARF11**	GRMZM2G056120	Forward GCACAAAAGTGTTTTTCACATTTC<br>Reverse TGTAAGTTGATCCTTGCTCCAATA
**ARF12**	GRMZM2G437460	Forward ACTCCTATTCCTGCACCTCATAAC<br>Reverse TTGTGAACTTCCAATAGGGTACAA
**ARF26**	GRMZM5G874163	Forward ATTTCAATAGTGACAGCACAAAGC<br>Reverse CTTCAGGAACTTAGCGTATGGAAT
**AGO7**	GRMZM5G892991	Forward CTGGCCTTGCTGTGTTAGGT<br>Reverse AGGTCAACATTGAGGGCAAG
**LBL1**	GRMZM2G020187	Forward CCATAGTGGCTGGGAAACCA<br>Reverse CTGGCCTTGCTGTGTTAGGT
**MIR390A**		Forward GCGAGGAGAAAGAAAGAGCCA<br>Reverse GATAGACAGAACCACGCCTCC
**MIR390B**		Forward AGATTGGAGCCACGAAGAGG<br>Reverse CGCTTCGGATCGATTCATCA
**Kn1**	GRMZM2G017087	Forward CTGAGTCTACCGGGCTTGAC<br>Reverse CTGACAAGACATGAGCCGTAC
**NAC116**	GRMZM2G0411746	Forward AACAGAGCACAAGACCAACC<br>Reverse ATGAGCTGCTGATGGTATACCG
**NAM1**	GRMZM2G393433	Forward ATTAACGGCCCCCTAGTTGC<br>Reverse TAGCTAGCATCCACTGCACG
**CUC3**	GRMZM2G430522	Forward CTGCATGCAAGAGCCG

		Reverse TGACATTAATCCGAAACAAACAGT
**WOX9A**	GRMZM2G133972	Forward CGTCGTCCCTACTCCTTCCT<br>Reverse GCATGTCCTGTGACCAGTGA
**WOX5A**	GRMZM2G478396	Forward AAGCAAAAGCAAACGGGAGG<br>Reverse CCTGTCCATGCAACGTTTGG
**WOX3A**	GRMZM2G122537	Forward CATGATCTCAGCTCGTCCCC<br>Reverse AAGTCCATACACACGGAGCG
**PIN1**<br>**PIN2**<br>**PIN3**	GRMZM2G098643<br>GRMZM2G074267<br>GRMZM2G149184	Forward GACTTCTACCACGTCATGACG<br>Reverse CTCGAACATGAACAGCATCAGC
**18S**		Forward TCC TAT TGT TGG CCT TCGG<br>Reverse TCC TTG GCA AAT GCT TTCGC
**tasiR-ARFbD5**	Primer stem loop	GTC GTA TCC AGT GCA GGG TCC GAG GTA TTC GCA CTG GAT ACG ACT TGG GT
	Forward primer	CGT CGC GTC TTG ACC TTG TAA G
**tasiR-ARFbD5**	Primer stem loop	GTC GTA TCC AGT GCA GGG TCC GAG GTA TTC GCA CTG GAT ACG ACA AAG GT
	Forward primer	CGG CGC TCT TGA CCT TGC
**tasiR-ARFg**	Primer stem loop	GTC GTA TCC AGT GCA GGG TCC GAG GTA TTC GCA CTG GAT ACG ACA AAG TC
	Forward primer	GAC CGG TTC TTG ACC TTG C
**Zma-mir390a-5p**	Primer stem loop	GTC GTA TCC AGT GCA GGG TCC GAG GTA TTC GCA CTG GAT ACG ACG GCG CT
	Forward primer	TCT GCG AAG CTC AGG AGG GAT
**U6 snRNA**	Primer stem loop	GTG CAG GGT CCG AGGTTT TGG ACC ATT TCT CGAT
	Forward primer	GGA ACG ATA CAG AGA AGATTA GCA

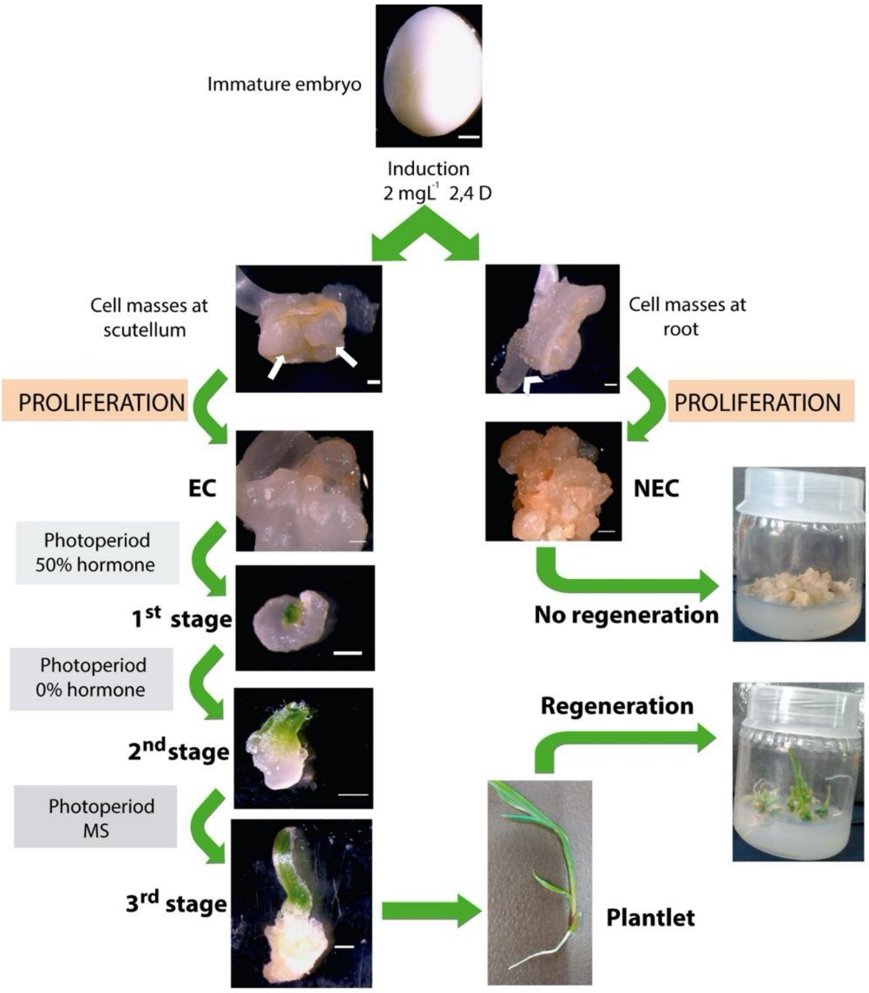

Figure S1. *In vitro* maize regeneration process. Maize immature embryos at 15-18 days after pollination were used as explant. Upon induction using N6PI medium, root protruded from embryo and two type of cell masses were established: one at the scutellum side (**arrow**) and the other localized at the side of the emerged root with waterish appearance (**arrowhead**). The scutellum-derived callus was named embryogenic callus (EC) and it was friable, compact and translucent. The callus that was formed from root was watery yellowish to brownish, not friable and it was called non-embryogenic callus (NEC). Both calli types were tested for plant regeneration, but only EC was able to regenerate plantlets. Plant regeneration was achieved via a gradual hormone reduction and exposure to photoperiod. After two weeks on N6P medium with half concentration of hormones, the initial evidence of regeneration was the formation of green spots at the callus surface; these were established as the first stage of development. Two additional weeks on N6P medium without hormones, the second stage of regeneration was stablished by presence of leaf-primordium-like structures. Upon two further weeks on MS medium, the third stage was recognized by the formation of a leaf with the typical parallel veins, but still adhered to callus, without the formation of roots. After a subsequent passage of regenerating tissues on MS medium, whole plantlets were established with emerging roots and some fully developed leaves.

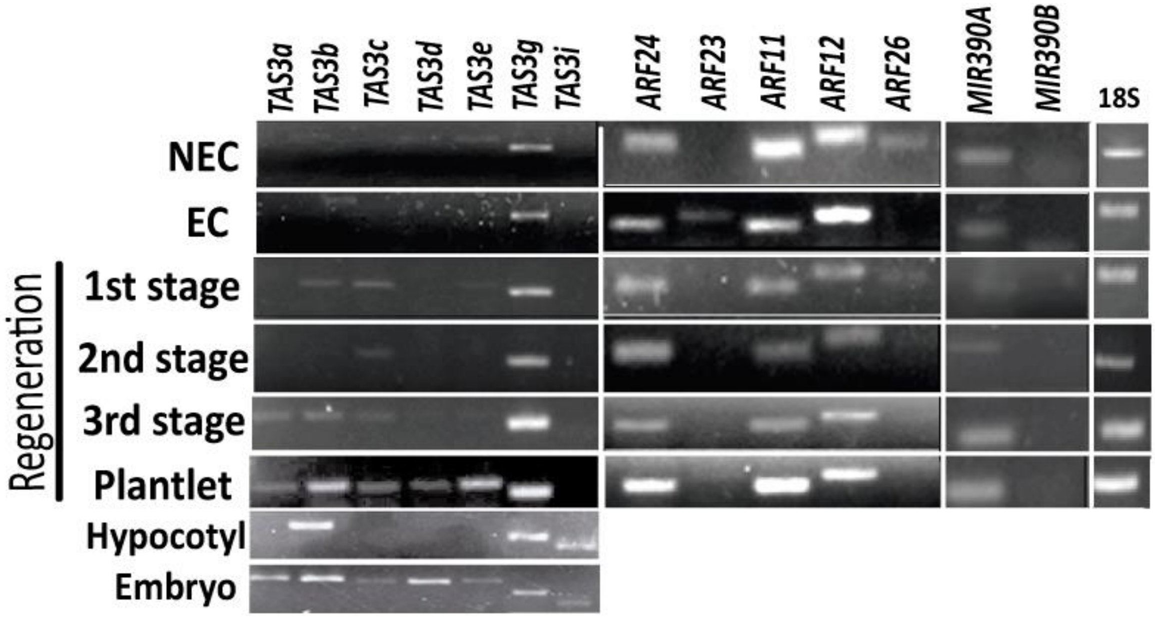

Figure S2. *TAS3, ZmARF* class 3/4, and *MIR390* transcript analysis during *in vitro* maize plant regeneration. The presence of transcripts originated from seven *TAS3*, five *ZmARF* from class 3/4 family members and two *MIR390* genes was evaluated by end-point RT-PCR in different tissues. The 18S rRNA was used as loading control. Embryogenic callus (EC); Non-embryogenic callus (NEC); Regeneration stages from EC under photoperiod were as follows: (1st stage) N6 with half-hormone depletion; (2nd stage) N6 with complete hormone depletion, (3rd stage) MS, (Plantlet) MS.

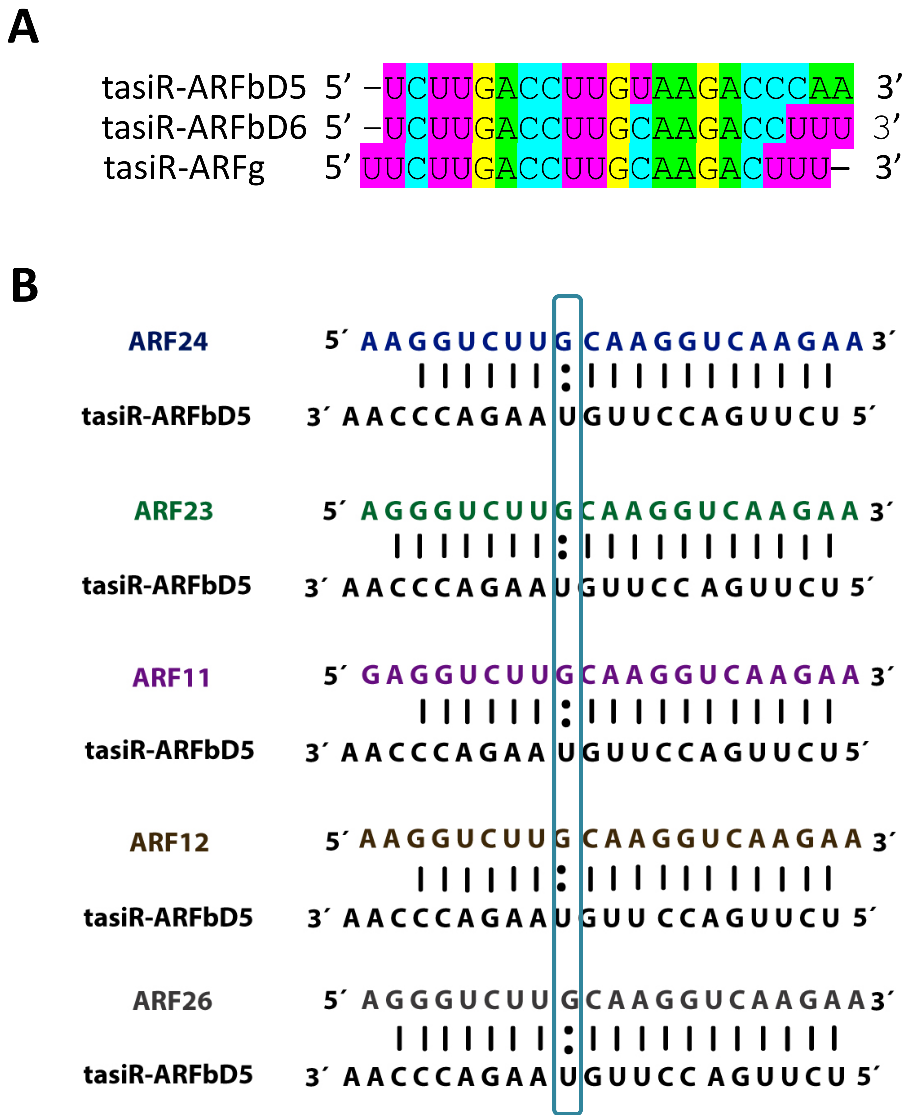

Figure S3. tasiR-ARF and ARF3-like *ZmARF* target sequences. A. Alignment of the three tasiR-ARFs analyzed in this study. **B.** Base pairing between tasiR-ARFbD5 and its target site in *ZmARF* transcripts. The highlighted G:U pair corresponds to a canonical G-C pair for tasiR-ARFbD6 and tasiR-ARFg.

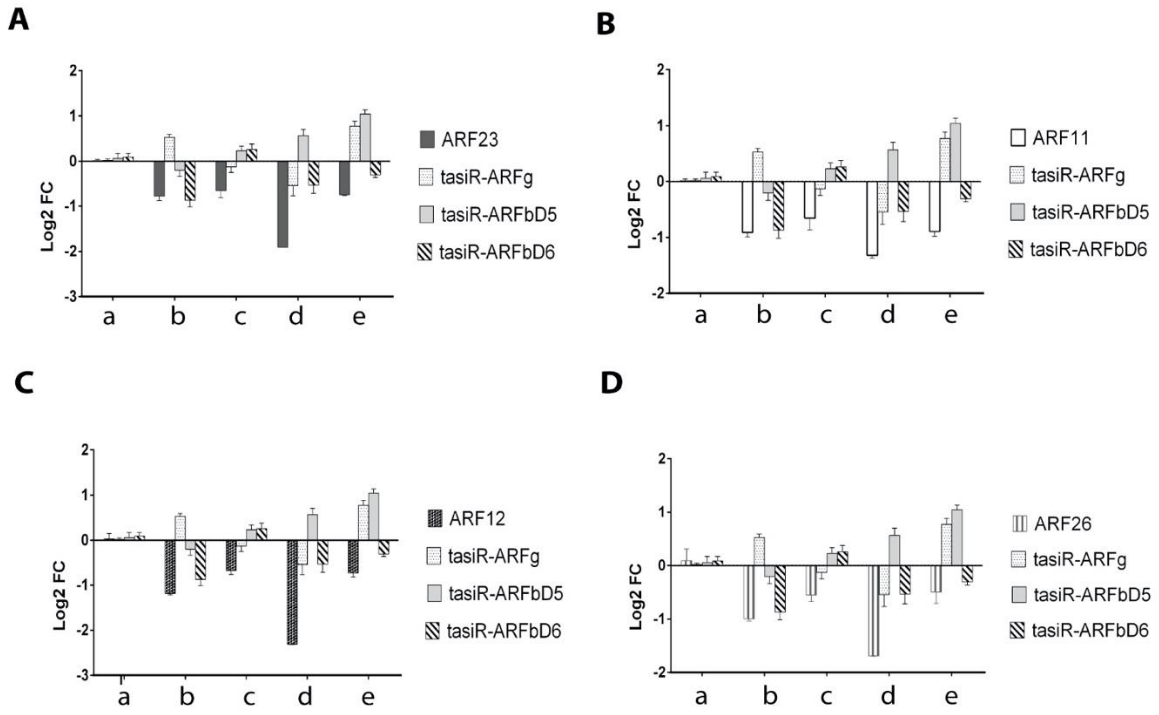

Figure S4. Stage-dependent inverse correlation between tasiR-ARFs and *ZmARF* targets. Inverse Correlation (IC) between three tasiR-ARFs and target *ZmARFs* at each regeneration stage was represented by log2 fold change (Log2FC) with respect to the starting material (EC). (a) EC, (b) 1st stage, (c) 2nd stage, (d) 3rd stage, (e) plantlet. **A**. IC for *ZmARF23* **B**. IC for *ZmARF11* **C**. IC for *ZmARF12*. **D.** IC for *ZmARF26*.

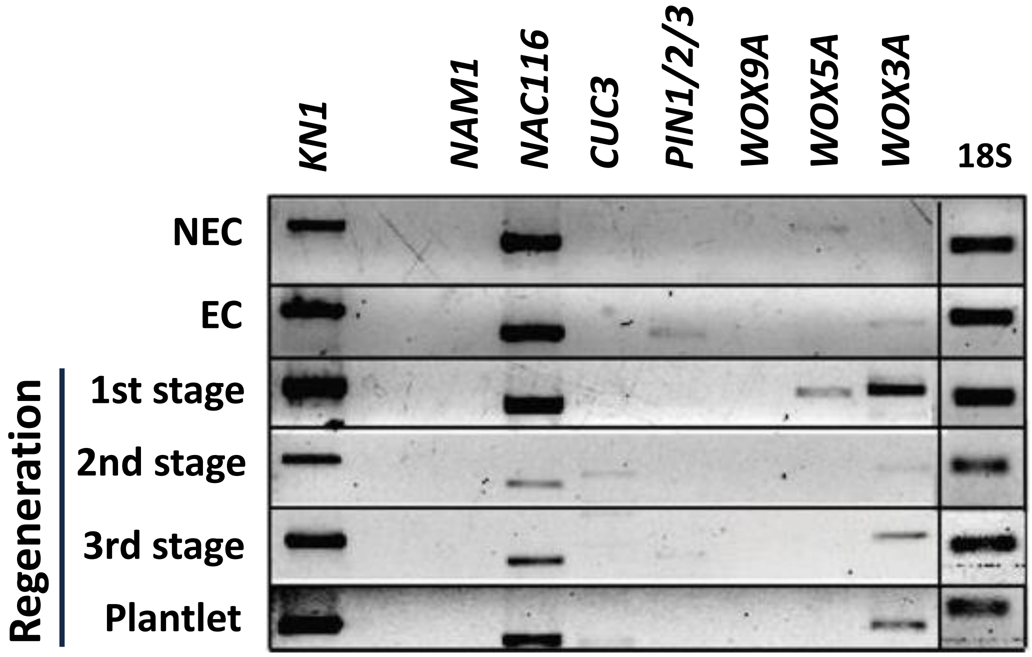

Figure S5. Transcript analysis of genes involved in maize SAM and leaf establishments during *in vitro* maize plant regeneration. Transcript levels were evaluated by end-point RT-PCR in different tissues. The 18S rRNA was used as loading control. (*KN1*) KNOTTED1, (*NAM1*) NO APICAL MERISTEM 1, (*NAC116*) NAC type transcription factor 116, (*CUC3*) CUP-SHAPED COTYLEDON 3, (*PIN1/2/3*) PINHEAD 1, 2 or 3, (*WOX9A*, *WOX5A*, *WOX3A*) WUSCHEL-related Homeobox genes. Embryogenic callus (EC); Non Embryogenic Callus (NEC) and regeneration stages under photoperiod were as follows: (1st stage) N6 with half-hormone depletion; (2nd stage) N6 with complete hormone depletion, (3rd stage) MS, (Plantlet) full leaves and rooting.
